# Anticoagulation and Arterial Thrombotic Events in Ambulatory Patients With Cancer: A Bayesian Re-Analysis and Perspective

**DOI:** 10.1016/j.jaccao.2023.09.005

**Published:** 2023-12-19

**Authors:** Ahmed Sayed, Daniel Addison

We read with great interest the meta-analysis by Xu et al^1^ on using anticoagulant agents to prevent arterial thrombotic events (ATEs) in ambulatory patients with cancer. We congratulate the investigators on this important analysis. However, we believe that their statement regarding anticoagulation’s effect on ATEs, “Anticoagulant use was not associated with risk reduction in ATE,” is worth revisiting.

**Figure 1:**
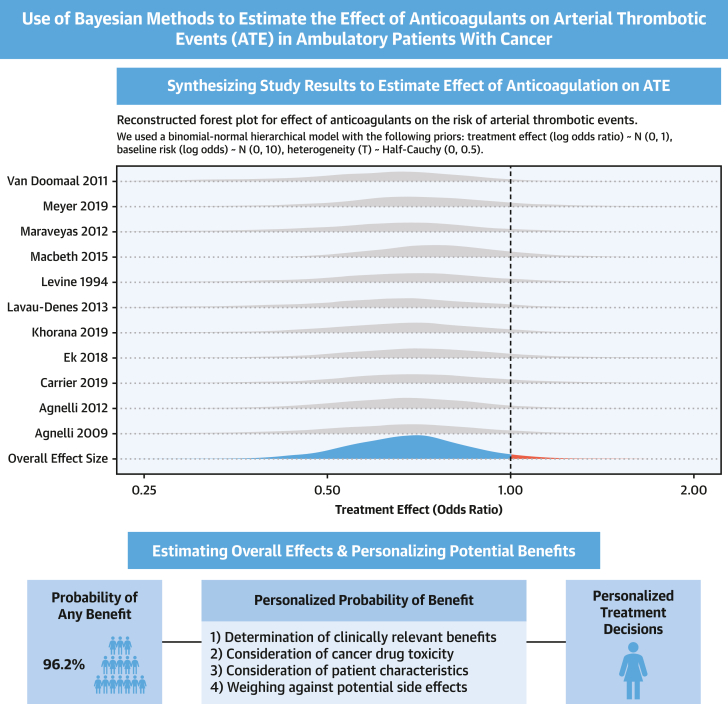
Use of Bayesian Methods for Estimation of Treatment Effects and Personalized Treatment Decisions The top panel shows the model type and priors we used to synthesize study estimates and calculate an overall (pooled) treatment effect. The bottom panel shows the probability that anticoagulation reduces the risk for arterial thrombotic events (ATEs) and how this probability can be tailored to each individual patient.

This statement rests on a risk ratio of 0.73 (95% CI: 0.50-1.04), which, although statistically nonsignificant, is suggestive of a reduction. Using a Bayesian approach with selected informative priors, we estimate a 96.2% probability that anticoagulation reduces ATEs (Figure 1). The selected priors used are shown in the figure and the code is publicly available.^2^

Using Bayesian methods, one can also calculate the probability of clinically relevant risk reductions. Two steps are needed. First, we must define the relevant magnitude of risk reduction. This varies on the basis of patient preference, resource availability, and side effect considerations (ie, major bleeding). For demonstrative purposes, let us presume that a 1% absolute reduction over 1 year is relevant.

Second, we must define the baseline risk. Let us presume that we will target a high-risk population, such as those treated with vascular endothelial growth factor–targeted tyrosine kinase inhibitors. An Italian study showed a 3.99% 1-year risk for ATEs.^3^ Using these data, we calculate a 67% probability that anticoagulant agents result in a relevant risk reduction (≥1%) of ATEs.

For a given relevant risk reduction, different baseline risks may result in different conclusions. In an Austrian cohort receiving immune checkpoint inhibitors, the 1-year risk for ATEs was 1.3%.^4^ With this baseline risk, the probability of a meaningful reduction becomes about 0%. Because baseline risk differs by the type of cancer treatment and individual characteristics, patient-level tailoring is necessary.

The decision to use preventive cardiovascular measures in patients with cancer must consider the magnitude of meaningful risk reductions and the patients’ baseline risk. Different preferences and baseline risks entail different decisions. Bayesian approaches offer another way to calculate benefit by calculating the probability that using a treatment will yield a meaningful risk reduction according to each patient’s baseline risk.
